# Screening is not associated with reduced incidence of gonorrhoea or chlamydia in men who have sex with men (MSM); an ecological study of 23 European countries

**DOI:** 10.12688/f1000research.17955.2

**Published:** 2019-09-12

**Authors:** Chris Kenyon

**Affiliations:** 1 University of Cape Town, Cape Town, South Africa, 7925, South Africa; 2 Institute of Tropical Medicine, Antwerp, Antwerp, 2000, Belgium

**Keywords:** Gonorrhoea, chlamydia, MSM, STI screening, PrEP, antimicrobial resistance

## Abstract

**Background: **Increasing rates of antimicrobial resistance has motivated a reassessment of if intensive screening for gonorrhoea and chlamydia is associated with a reduction in the prevalence of these infections in men who have sex with men (MSM).

**Methods: **Spearman’s correlation was used to evaluate the country-level correlation between the intensity of self-reported sexual transmitted infection (STI) screening in MSM (both anal and urethral screening, taken from a large internet survey of MSM) and the incidence (taken from ECDC surveillance figures) and prevalence (taken from a literature review of studies estimating prevalence in MSM attending STI clinics) of gonorrhoea and chlamydia.

**Results:** The intensity of both anal and genital screening was found to be positively associated with country level gonorrhoea incidence rates (rho 0.74; p=0.0004; rho=0.73; p=0.0004, respectively) and Ct incidence rates (rho 0.71; p=0.001; rho=0.78; p=0.0001, respectively). No associations were found between anal or genital screening intensity and Ng prevalence in clinic populations (Table 2).

**Conclusions: **We found no evidence of a negative association between screening intensity and the prevalence of gonorrhoea or chlamydia in MSM. Randomized controlled trials are urgently required to evaluate if the high antimicrobial exposure resulting from intensive screening programmes is justified.

## Introduction

There have been large increases in antimicrobial resistance in a number of sexual transmitted infections (STI) in the recent past. There are serious concerns that both *Neisseria gonorrhoeae* (Ng) and *Mycoplasma genitalium* may become untreatable in the not too distant future ^1, 2^. For both these bacteria as well as macrolide resistance in *Treponema pallidum,* AMR has frequently first emerged in populations with a combination of high antimicrobial consumption and dense sexual networks ^3, 4^. HIV preexposure prophylaxis (PrEP) cohorts have dense sexual networks and the intense screening STI typically practiced translates into high antimicrobial exposures ^5, 6^. Three-monthly, 3-site Ng/ *Chlamydia trachomatis* (Ct) screening for example translates into macrolide exposures of around 4400 standard units/1000 population/year, which is many times higher than levels associated with the induction of macrolide resistance in a range of bacteria including *T. pallidum* and Ng ^7, 8^. These findings have led a number of authors to review the evidence to support Ng/Ct screening in men who have sex with men (MSM) PrEP populations.

The US Preventive Task Force, concluded that there is insufficient evidence to advocate for or against screening for Ng in men, including MSM ^9^. In a systematic review conducted to inform these guidelines, the authors found no randomised, controlled trials or controlled observational studies that assessed the utility of NG screening in men ^10^. In a systematic review of observational studies, we found no evidence that even the most intense Ng/Ct screening such as screening 100% of PrEP cohorts every 3 months was associated with a decline in the prevalence of these infections ^11^. Others have argued that this lack of an effect was because the PrEP recipients were having sex with (and being reinfected by) people who were not being screened ^12^. This generates the hypothesis that we test in this paper that populations where a high proportion of MSM are screened for Ng/Ct will have a lower prevalence of these infections than populations with less screening. We test this hypothesis in European countries because the intensity of STI screening varies widely here and data for screening and prevalence estimates were available. 

## Methods

### Data sources

***STI screening intensity***. Country level STI screening prevalence were obtained from the European MSM Internet Survey (EMIS), which was an internet-based survey of over 160 000 MSM from 38 countries living in Europe ^13^. The survey was conducted between June and August 2010. In the section where participants were asked about STI testing in the past 12 months, they were asked 3 questions that are relevant to Ng/Ct screening: Did you provide a urine sample for STI screening? Was urethral swab inserted into your penis for STI screening? Was a swab inserted into your anus for STI screening? EMIS combined the results from the first two questions into one variable reporting the proportion of respondents reporting ‘urethral STI screening’ – via either urine or urethral swab. The third question provided the proportion with ‘anal STI screening’. Typically, these urethral and anal samples are tested for Ng/Ct.

***Ng/Ct prevalence/incidence***.

**1.**  National Ng and Ct incidence estimates for men in 2010 were taken from European Centre for Disease Prevention and Control (ECDC) figures ^14^. These incidence estimates are based on national surveillance systems. The ECDC does not provide incidence estimates separately for MSM and thus we used the estimates for all men. MSM do however constitute a high proportion of diagnoses in all men ^14^.
**2.**  Systematic review of Ng/Ct prevalence in MSM
  Ng/Ct prevalence estimates for MSM were taken from a published literature review of pharyngeal and anorectal Ng and Ct prevalence estimates in MSM (and other populations) ^15^. All studies listed in PubMed reporting prevalence of extragenital Ng and Ct in MSM up to 1 December 2015 were included. A total of 53 studies were included from countries around the world. Of these 18 were from 6 European countries ( Table 1). For the four European countries with more than one study we selected the study reporting prevalence estimates from 2010 or as soon after this year as possible. All selected studies were prevalence estimates established by Nucleic Acid Amplification Testing of MSM clients attending STI clinics.

**Table 1.** Prevalence of sexual transmitted infection (STI) screening, STI incidence and prevalence in European countries with available data.

Country	Screening Prevalence in 2010 (%)		STI Incidence in men 2010 (cases/100 000/ year)		STI Prevalence in MSM attending STI clinics (%)						
					*N. gonorrhoeae*			*C. trachomatis*			Reference
	Urethral	Anal	Ng	Ct	Urethral	Rectal	Pharyngeal	Urethral	Rectal	Pharyngeal	
**Bulgaria**	39.2	10.0	2.7	.5							
**Cyprus**	59.9	17.3		.5							
**Czech Rep.**	67.2	19.6	10.4								
**Germany**	56.6	20.6			1.9	4.6	5.5	3.4	8	1.5	doi.org/10.1136/ sextrans-2012-050929
**Denmark**	71	40.0	13.2	383.7				2.6	2.6	0	doi.org/10.1136/ sti.73.6.493
**Estonia**	58.4	14.2	6.5	40.5							
**Greece**	41.4	11.8	4.6	1.4							
**Spain**	52	15.2					9.5				doi.org/10.1177/095646 2413486455
**Finland**	89	37.9	7.2	201							
**Ireland**	91	67.2	20.5	104.7	0	4.1	3.3	1.7	6.6	.8	
**Lithuania**	63.6	13.3	18.3	15.7							
**Luxembourg**	41.9	9.1	1.2	0							
**Latvia**	64	14.9	26	33.8							
**Malta**	85	70.6	20.9	37.5							
**Netherlands**	87	63.0			3.4	5.5	3.9	4.3	10.1	1.7	doi.org/10.1177/095646 2414521165
**Norway**	84	47.2	15	353.8							
**Poland**	37	8.9	1.5	2.2							
**Portugal**	68.9	9.5	1.5								
**Romania**	45	6.5	4.1	.7							
**Sweden**	92	59.0	13.3	333.3							
**Slovenia**	50	29.0	4.1								
**Slovakia**	60	16.9	3.6								
**United** **Kingdom**	94	69.7	42.2		4.7	9	5.2	5.3	6.5	2.2	doi.org/10.1258/ ijsa.2012.011378

*N. gonorrhoeae* - *Neisseria gonorrhoeae*, *C. trachomatis* - *Chlamydia trachomatis*

### Data analysis

In all analyses the correlation between screening intensity and Ng/Ct prevalence/incidence was tested using Spearman’s correlation. The statistical analyses were performed in STATA 13.

## Results

### STI screening

The proportion of respondents in each of the 23 countries reporting anal STI screening varied widely from 6.5 to 70.6% to (median 17.3%, IQR 11.8–47.1; Table 1). Likewise, there were large variations in the proportion reporting genital STI screening (range 37.0 to 94.0%, median 63.6 IQR 50.0–85.0%). There was a strong correlation between the proportions reporting anal and genital STI screening (rho=0.81; p<0.0001).

### Incidence of Ng and Ct based on ECDC estimates

For 19 countries with data, the incidence of Ng for men in 2010 ranged between 1.2 and 42.2 cases per 100 000 men per year (median 7.2, IQR 3.6–18.3). There was an even wider distribution in estimated Ct incidence for the 18 countries with data (range 0 to 383.7, median 24.8, IQR 1.3–201).

### Prevalence of Ng/Ct in MSM based on STI clinic attendees

There was less variance in the prevalence estimates of Ng and Ct in MSM ( Table 1). Rectal Ng: median 5.5%, IQR 4.6–7; pharyngeal Ng: median 5.4%, IQR 3.9–6.5; urethral Ng: median 1.9%, IQR 1–3.4. Rectal Ct: median 7.3%, IQR 6.5–10.0; pharyngeal Ct: median 1.3, IQR 0.8–1.7; urethral Ct: median 3, IQR 2.5–5.3; Table 1.

### Correlation between screening intensity and Ng/Ct incidence/prevalence

**Figure 1.**
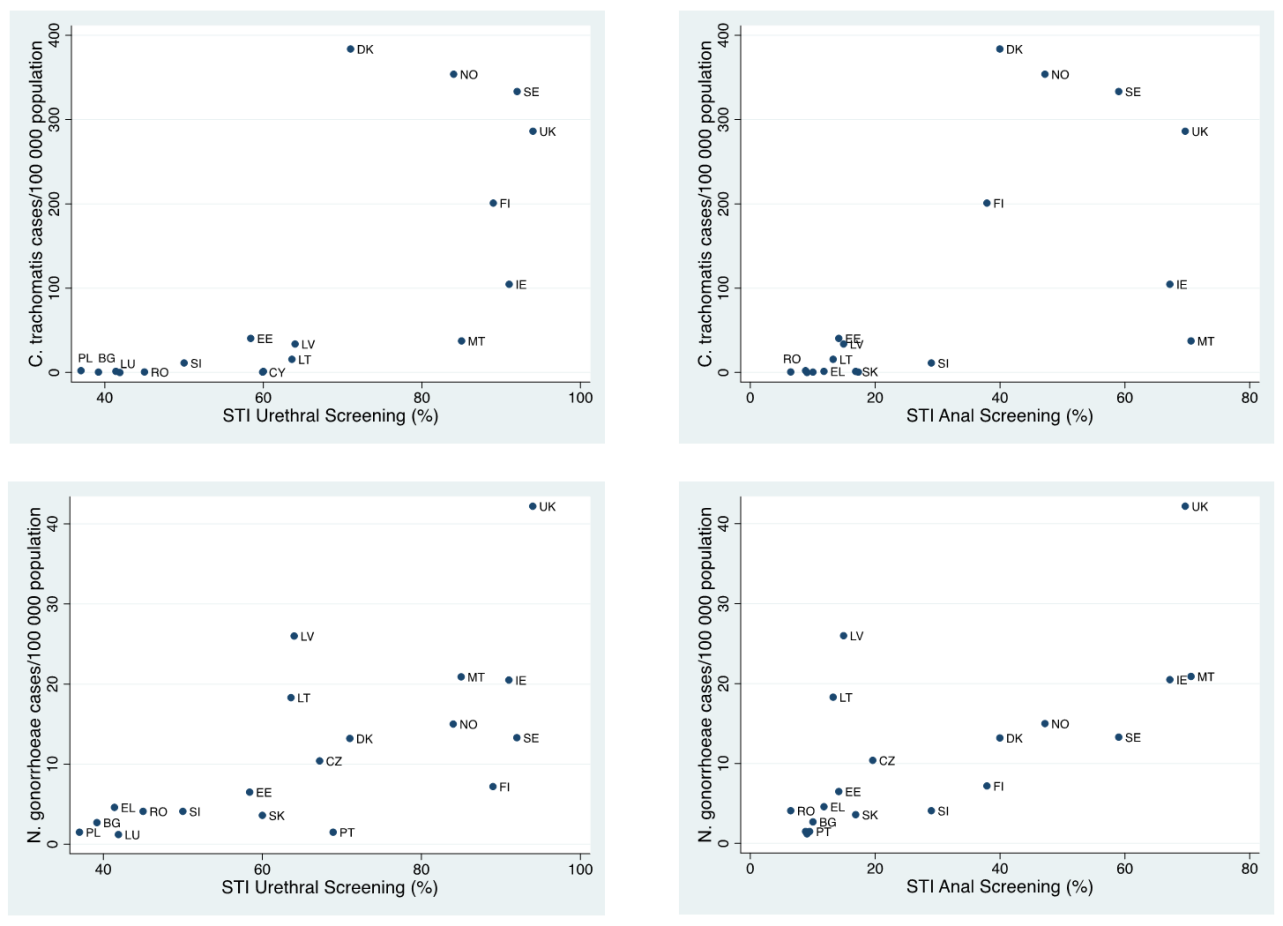
Scatter plots of country-level association between self-reported anal and urethral sexual transmitted infection (STI) screening intensity in men who have sex with men (MSM) and incidence of *Chlamydia trachomatis* and *Neisseria gonorrhoeae* in men in European countries in 2010. Country designations: AT, Austria; BE, Belgium; CZ, Czech Republic; DE, Germany; DK, Denmark; EL, Greece; ES, Spain; FI, Finland; FR, France; HR, Croatia; HU, Hungary; IE, Ireland; IT, Italy; LU, Luxembourg; LV, Latvia; NL, the Netherlands; PT, Portugal; SE, Sweden; SI, Slovenia; SK, Slovakia; UK, United Kingdom.

The intensity of both anal and genital screening was found to be positively associated with country level Ng incidence rates (rho 0.74; p=0.0004; rho=0.73; p=0.0004, respectively) and Ct incidence rates (rho 0.71; p=0.001; rho=0.78; p=0.0001, respectively; Figure 1).

No associations were found between anal or genital screening intensity and Ng or Ct prevalence in clinic populations ( Table 2).

**Table 2.** Spearman’s correlation between prevalence of sexual transmitted infection (STI) screening (anal and urethral) and prevalence of *Neisseria gonorrhoeae* and *Chlamydia trachomatis* (pharyngeal, rectal and urethral). All P-values were greater than 0.1.

STI prevalence	Anal testing	Urethral testing
*N. gonorrhoeae*		
**Pharyngeal**	-0.70	-0.70
**Rectal**	0.40	0.40
**Urethral**	0.40	0.40
*C. trachomatis*		
**Pharyngeal**	0.50	0.50
**Rectal**	-0.20	-0.20
**Urethral**	0.30	0.30

## Discussion

The prevalence of Ng and Ct has been increasing in MSM populations in a number of countries ^16, 17^. Intensified screening in MSM would be one way to reduce the incidence and prevalence of these infections. In this analysis, we did not find evidence of a negative correlation between the intensity of STI screening in MSM and the incidence/prevalence of Ng/Ct. Instead, we found evidence of a positive association between the intensity of screening in MSM and the estimated incidence rate for men. This positive association may be explained by the fact the incidence estimates are influenced by the intensity of screening – countries with more intensive screening programmes would be expected to diagnose more asymptomatic Ng and Ct infections which lead to higher incidence estimates. This could also be considered a form of reverse causation: the higher prevalence of Ng/Ct is the cause rather than the effect of more intensive screening.

To deal with this bias and the fact that the ECDC Ng/Ct incidence estimates do not provide incidence estimates for MSM, we also evaluated the association between screening intensity and Ng/Ct prevalence in MSM attending STI clinics. Here we found no evidence of an association between screening intensity and prevalence.

These findings are open to a number of interpretations. Firstly, screening intensity may be negatively associated with Ng/Ct rates but we missed this association due to methodological issues. Our estimates of screening intensity were based on a single cross-sectional source. Although EMIS had a large sample size and the accuracy of its prevalence estimates for other variables has been validated in other studies ^13, 18^, these screening estimates may be inaccurate and may have changed over time. As noted above, the STI incidence estimates were for all men and were likely strongly influenced by practices such as screening intensity, access to health care and accuracy of national case reporting. The STI prevalence estimates in MSM were all taken from men attending STI clinics and thus are likely higher than general populations of MSM. The study design of each of the 6 studies contributing Ng and CT prevalence estimates differed somewhat further limiting the extent to which correlations could be assessed between screening intensity and prevalence across these studies. We could find no comparable data on the prevalence of Ng or Ct in general MSM populations.

Alternatively, screening intensity as measured may not be associated with reduced Ng/Ct rates in MSM. Randomized controlled trials of the efficacy of screening for Ct in women on the prevalence of Ct have produced equivocal results ^19– 22^. Although no RCTs have been conducted in MSM, a systematic review of observational studies revealed that Ng/Ct screening, even when conducted at 3-sites every 3-months, was not associated with reductions in the prevalence of Ng or Ct ^11^. If we consider Ng, numerous aspects of the way it circulates in contemporaneous populations of MSM may explain why screening has little or no effect on prevalence. Symptomatic disease is thought to typically occur soon (2–21 days) after infection and if symptoms do not develop the infection (particularly in the pharynx and rectum) tends to persist in a low abundance, low infectious state for up to 6 months ^23^. Highly exposed individuals develop a type-specific immunity, but this immunity is largely ineffective in low exposure individuals ^23, 24^. As a result, the vast majority of Ng infections are asymptomatic and self-limiting in MSM PrEP populations ^23, 25^. Similar considerations apply to Ct. In the case of Ct there is however better evidence that treatment of Ct results in “arrested immunity” and thereby paradoxically increases the probability of reinfection ^26, 27^. If screening results in ‘arrested immunity’ it may paradoxically increase Ng/Ct prevalence/symptomatic disease. The sexual networks of PrEP recipients are very dense (up to a mean of 18 partners per 3 months ^28^) and this is responsible for generating the high prevalences of Ng and Ct ^5^. Removing individuals piecemeal from this network for screening and treating has no effect on the underlying determinant of high prevalence. As a result, the probability of reinfection and prevalence remaining high.

Mathematical models of Ng and Ct transmission in European countries like Belgium have thus found that the sexual network of MSM was so dense that current levels of Ng screening were having little to no effects on Ng prevalence ^29^. In contrast, a modelling study from the United States found that 6-monthly screening of an expanded number of PrEP recipients could avert 40% of Ng and Ct infections ^30^. This study did not however model pharyngeal transmission of Ng (which plays a major role in transmission) and did not model the impact of immunity or Ng’s ability to adapt to antibiotic pressure. These omissions may explain the discrepancy between its prediction, and our and the earlier systematic review of observational studies ^11^.

Based on the findings of this study and those reviewed here we conclude that we can still not exclude the possibility that intense screening (at least 3-site, 3-monthly) may have a small to moderate influence on Ng/Ct prevalence in MSM. Randomized controlled trials are urgently required to test this hypothesis. In the interim, given the mounting evidence that Ng/Ct screening does not have a large effect on prevalence but does result in high levels of antimicrobial exposure, consideration should be given to reducing the intensity or stopping Ng/Ct screening in MSM in a phased and controlled manner that allows a detailed evaluation of the risks and benefits of screening.

## Data availability

### Underlying data

All data underlying the results are available as part of the article and no additional source data are required.

## References

[1] Eyre DW Sanderson ND Lord E: Gonorrhoea treatment failure caused by a *Neisseria gonorrhoeae* strain with combined ceftriaxone and high-level azithromycin resistance, England, February 2018. *Euro Surveill.* 2018;23(27):1800323. 10.2807/1560-7917.ES.2018.23.27.1800323 29991383 PMC6152157

[2] Bradshaw CS Horner PJ Jensen JS: Syndromic management of STIs and the threat of untreatable *Mycoplasma genitalium*. *Lancet Infect Dis.* 2018;18(3):251–2. 10.1016/S1473-3099(18)30080-X 29485089

[3] Lewis DA: The role of core groups in the emergence and dissemination of antimicrobial-resistant *N gonorrhoeae*. *Sex Transm Infect.* 2013;89 Suppl 4:iv47–51. 10.1136/sextrans-2013-051020 24243880

[4] Kenyon C: Prevalence of macrolide resistance in *Treponema pallidum* is associated with macrolide consumption. *J Med Microbiol.* 2018. 10.1099/jmm.0.000885 30520715

[5] Kenyon C Schwartz IS: Effects of Sexual Network Connectivity and Antimicrobial Drug Use on Antimicrobial Resistance in *Neisseria gonorrhoeae*. *Emerg Infect Dis.* 2018;24(7):1195–1203. 10.3201/eid2407.172104 29912682 PMC6038757

[6] Kenyon C: Risks of Antimicrobial Resistance in *N. gonorrhoeae* Associated with Intensive Screening Programs in Pre-Exposure Prophylaxis Programs. *Clin Infect Dis.* 2018;67(1):154–5. 10.1093/cid/ciy048 29370373

[7] Kenyon C: We need to consider collateral damage to resistomes when we decide how frequently to screen for chlamydia/gonorrhoea in preexposure prophylaxis cohorts. *AIDS.* 2019;33(1):155–7. 10.1097/QAD.0000000000002020 30234609

[8] Kenyon C Buyze J Wi T: Antimicrobial Consumption and Susceptibility of *Neisseria gonorrhoeae*: A Global Ecological Analysis. *Front Med (Lausanne).* 2018;5:329. 10.3389/fmed.2018.00329 30538989 PMC6277557

[9] LeFevre ML; U.S. Preventive Services Task Force: Screening for Chlamydia and gonorrhea: U.S. Preventive Services Task Force recommendation statement. *Ann Intern Med.* 2014;161(12):902–10. 10.7326/M14-1981 25243785

[10] Zakher B Cantor AG Pappas M: Screening for gonorrhea and Chlamydia: a systematic review for the U.S. Preventive Services Task Force. *Ann Intern Med.* 2014;161(12):884–93. 10.7326/M14-1022 25244000

[11] Tsoumanis A Hens N Kenyon CR: Is Screening for Chlamydia and Gonorrhea in Men Who Have Sex With Men Associated With Reduction of the Prevalence of these Infections? A Systematic Review of Observational Studies. *Sex Transm Dis.* 2018;45(9):615–622. 10.1097/OLQ.0000000000000824 29485537

[12] Ridpath AD Chesson H Marcus JL: Screening Peter to Save Paul: The Population-Level Effects of Screening Men Who Have Sex With Men for Gonorrhea and Chlamydia. *Sex Transm Dis.* 2018;45(9):623–5. 10.1097/OLQ.0000000000000892 29994935 PMC6086737

[13] The EMIS Network: The European MSM Internet Survey (EMIS) Community Report. Stockholm: European Centre for Disease Prevention and Control, 2013 Contract No.: 01/04/2014.

[14] European Centre for Disease Prevention and Control: Sexually transmitted infections in Europe 1990–2010. Stockholm: ECDC.2012 Reference Source

[15] Chan PA Robinette A Montgomery M: Extragenital Infections Caused by *Chlamydia trachomatis* and *Neisseria gonorrhoeae*: A Review of the Literature. *Infect Dis Obstet Gynecol.* 2016;2016:5758387. 10.1155/2016/5758387 27366021 PMC4913006

[16] Callander D Guy R Fairley CK: Gonorrhoea gone wild: rising incidence of gonorrhoea and associated risk factors among gay and bisexual men attending Australian sexual health clinics. *Sex Health.* 2018. 10.1071/SH18097 30409244

[17] Unemo M Bradshaw CS Hocking JS: Sexually transmitted infections: challenges ahead. *Lancet Infect Dis.* 2017;17(8):e235–e79. 10.1016/S1473-3099(17)30310-9 28701272

[18] Marcus U Hickson F Weatherburn P: Estimating the size of the MSM populations for 38 European countries by calculating the survey-surveillance discrepancies (SSD) between self-reported new HIV diagnoses from the European MSM internet survey (EMIS) and surveillance-reported HIV diagnoses among MSM in 2009. *BMC Public Health.* 2013;13:919. 10.1186/1471-2458-13-919 24088198 PMC3850943

[19] Andersen B van Valkengoed I Sokolowski I: Impact of intensified testing for urogenital *Chlamydia trachomatis* infections: a randomised study with 9-year follow-up. *Sex Transm Infect.* 2011;87(2):156–61. 10.1136/sti.2010.042192 21097811

[20] van den Broek IV van Bergen JE Brouwers EE: Effectiveness of yearly, register based screening for chlamydia in the Netherlands: controlled trial with randomised stepped wedge implementation. *BMJ.* 2012;345:e4316. 10.1136/bmj.e4316 22767614 PMC3390168

[21] Low N Redmond S Uusküla A: Screening for genital chlamydia infection. *Cochrane Database Syst Rev.* 2016; (9):CD010866. 10.1002/14651858.CD010866.pub2 27623210 PMC6457643

[22] Hocking JS Temple-Smith M Guy R: Population effectiveness of opportunistic chlamydia testing in primary care in Australia: a cluster-randomised controlled trial. *Lancet.* 2018;392(10156):1413–22. 10.1016/S0140-6736(18)31816-6 30343857

[23] Hook EW Handsfield H: Gonococcal infections in the adult.In: Holmes KK, editor. *Sexually transmitted diseases.*3rd ed. New York: McGraw-Hill, Health Professions Division;1999; xxiii, 1454, 118.

[24] Plummer FA Simonsen JN Chubb H: Epidemiologic evidence for the development of serovar-specific immunity after gonococcal infection. *J Clin Invest.* 1989;83(5):1472–6. 10.1172/JCI114040 2496142 PMC303849

[25] Fairley CK Hocking JS Zhang L: Frequent Transmission of Gonorrhea in Men Who Have Sex with Men. *Emerg Infect Dis.* 2017;23(1):102–4. 10.3201/eid2301.161205 27983487 PMC5176237

[26] Geisler WM Lensing SY Press CG: Spontaneous resolution of genital Chlamydia trachomatis infection in women *and protection from reinfection*. *J Infect Dis.* 2013;207(12):1850–6. 10.1093/infdis/jit094 23470847 PMC3654745

[27] Omori R Chemaitelly H Althaus CL: Does infection with *Chlamydia trachomatis* induce long-lasting partial immunity? Insights from mathematical modelling. *Sex Transm Infect.* 2019;95(2):115–121. 10.1136/sextrans-2018-053543 30181327 PMC6580764

[28] Grant RM Lama JR Anderson PL: Preexposure chemoprophylaxis for HIV prevention in men who have sex with men. *N Engl J Med.* 2010;363(27):2587–99. 10.1056/NEJMoa1011205 21091279 PMC3079639

[29] Buyze J Vanden Berghe W Hens N: Current levels of gonorrhoea screening in MSM in Belgium may have little effect on prevalence: a modelling study. *Epidemiol Infect.* 2018;146(3):333–338. 10.1017/S0950268818000092 29386078 PMC9134539

[30] Jenness SM Weiss KM Goodreau SM: Incidence of Gonorrhea and Chlamydia Following Human Immunodeficiency Virus Preexposure Prophylaxis Among Men Who Have Sex With Men: A Modeling Study. *Clin Infect Dis.* 2017;65(5):712–718. 10.1093/cid/cix439 28505240 PMC5848234

