# Supplementary material for: Screening is not associated with reduced incidence of gonorrhoea or chlamydia in men who have sex with men (MSM); an ecological study of 23 European countries
Source: F1000Res. 2019 Sep 12;8:160. Originally published 2019 Feb 6. [Version 2] doi: 10.12688/f1000research.17955.2 (PMC6733379; doi:10.12688/f1000research.17955.2)
Supplement: Peer review history

# Peer review: Reviewer response for version 2

**DOI:** 10.5256/f1000research.22633.r50515

**Published:** 2019-09-20

**Authors:** Mirjam Kretzschmar

**Affiliations:** 1 Julius Center for Health Sciences and Primary Care, University Medical Center, Utrecht, The Netherlands; 2 Centre for Infectious Disease Control, National Institute of Public Health and the Environment (RIVM), Bilthoven, The Netherlands

In this paper the author uses a combination of data sources to study the relationship of testing and treatment for gonorrhoea and chlamydia infections and the incidence and prevalence of these infections in MSM populations. The main finding is that screening rates and incidence are positively correlated. It is an interesting idea to analyse these ecological relationships, however, as the author also acknowledges, there are many caveats for the interpretation of the results. Ecological analyses are always fraught with difficulties, as there is no information on individual level on the relationship between variables. In the data sets used in this paper the difficulties are magnified by a number of properties of the data sets used, which are not sufficiently discussed in the paper. 

 Firstly, the EMIS data set: To understand the quality of the data about STI screening rates, one would need to know how representative EMIS respondents were for the general MSM population. How were EMIS respondents recruited, how does their sexual risk behaviour compare to other MSM study populations? Were there differences between countries participating in the EMIS survey in recruitment and representativeness? 

 Secondly, the ECDC surveillance data set: ECDC collects data from national surveillance systems of member states in their TESSy data base. The quality and completeness of national surveillance systems is very variable between countries, and depends strongly on health care systems in the countries. In addition, given that a large proportion of all chlamydia infections and a good proportion of gonorrhoea infections are asymptomatic, reporting of these infections in surveillance systems will always reflect mainly the health seeking behaviour of the population and quality of reporting, more than the incidence of an infection. Estimating incidence from these data is difficult if not impossible, and in my opinion these data cannot be interpreted as incidence. It is therefore very plausible that numbers of notified cases increase with screening rates, which is reflected in the positive correlations, but this does not give any information about possible impact of screening on incidence. ECDC has also published a review on the impact of screening on chlamydia prevalence in Europe, which may be worthwhile mentioning in this context ( ECDC 2014). 

 Thirdly, prevalence of STI in MSM who attend STI clinics: Again, it is difficult to say what these estimates mean for the prevalence in the general MSM population, as men attending STI clinics are a selected group with in general higher risk of acquiring STIs than average. What proportion of men attend STI clinics also depends on the health care system of a country and possible stigmatization of MSM in the society.  It is striking that prevalence estimates are   available mainly for North-Western European countries, which may be similar in their health care systems, and not for other European regions. How does the population of STI clinic patients compare with the participants of the EMIS survey in terms of sexual behaviour and health care uptake?

 This ecological analysis could be improved and would be more interesting, if it would include a more in-depth comparison of the study populations and a more extensive analysis of how data were collected and their possible biases.

I confirm that I have read this submission and believe that I have an appropriate level of expertise to confirm that it is of an acceptable scientific standard, however I have significant reservations, as outlined above.

# Peer review: Reviewer response for version 1

**DOI:** 10.5256/f1000research.19637.r53339

**Published:** 2019-09-03

**Authors:** Eline L Korenromp, Nico Nagelkerke

**Affiliations:** 1 Avenir Health, Geneva, Switzerland; 2 Independent, Innsbruck, Austria

General Comments:

 This is a useful study, well designed, analyzed, and written, ending in a well-reasoned call for improving the evidence base for gonorrhea and chlamydia screening programs.

 The discussion adds particular value, comparing and contrasting findings from empirical studies with predictions by modelling studies. This part could possibly be structured better (e.g. I found it confusing to see reference 11 discussed twice on page 6). The authors might add a general statement about the value of mathematical models to answer complex epidemiological questions such as this. For example " *This illustrates that mathematical models to assess health interventions may be wrong for reasons that are not always obvious, and underlines the importance of empirical evidence".*

 The specific comments below may serve to improve readability and interpretation.

 Methods:

 Page 3, right column, top: ‘ *A total of 53 studies were included of which 18 were from 6 European countries’*. I suppose the remaining 35 studies were from other European countries, or also non-European? Perhaps just drop ‘European’ from this sentence, so to avoid this confusion.

 While, according to reference 14, a large proportion of Gc/Ct diagnoses takes places in MSM, it is unlikely that this reflects actual incidence, especially with regard to Ct which is highly prevalent, often asymptomatically, in the heterosexual population (e.g. in adolescent girls). This may be worth mentioning (in Introduction or Discussion) – to put the importance of MSM in the overall epidemic in context.

 Results:

 Page 4: ‘No associations were found between anal or genital screening intensity and Ng prevalence in clinic populations’. You found the same for CT, right? Please add that.

 Discussion:

 Page 4: The authors offer as a possible explanation for the observed positive (and not, the hoped negative) association that screening makes people susceptible to reinfection. To me a more basic explanation in this ecological analysis based on cross-sectional data, is screening programs are likely targeted to regions and populations with high prevalence. Reverse causality: the high prevalence is likely the cause rather than the effect of the screening program being there.

 Page 5: ‘ *To deal with this bias … we also evaluated the association in MSM attending STI clinics’*: Not clear, the association between what and what? You mean, between national screening intensity and prevalence in MSM attending STI clinics? Please rephrase, and refer back to Table 2 here.

 Page 5: ‘ *As noted above, the STI incidence estimates were for all men and were likely strongly influenced by practices such as screening intensity’*. A more general limitation of these incidence estimates based on national surveillance data is that NG and Ct incidence is hardly measurable, and case notifications are no good indication of underlying incidence of these infections, which are most often asymptomatic and not presenting to clinics. Besides variations in screening intensity, variations across countries in health care access and population awareness of STIs contribute to varying case notification rates, which do not reflect true variations in underlying incidence, and so may bias (or at least dilute the power) of the ecological analysis.

 Page 6: ‘… *limiting the extent to which comparisons can be made between prevalence estimates’:*

 Please consider to rephrase as *‘… limiting the extent to which correlations could be assessed between screening intensity and prevalence across these studies’*

 Page 6: About the systematic review of observational studies, quoted as reference 11, could you summarize how this differs from your current study, in methodology, scope, populations covered and/or other aspect?

 Page 6: *‘These omissions may explain the discrepancy between its finding and that of the systematic review of…*’ This sentence may be clearer if written as: *‘… between its prediction, and our and the earlier systematic review of observational studies’.*

 Conclusion:

 The first concluding sentence is a perhaps optimistic twist to a negative finding, which may surprise some readers (such as me). It does serve as a good introduction to the authors’ call for randomized trials to test the impact of screening in MSM. For readability, the authors may consider to a few word changes: *‘Based on the findings of this study and those reviewed we conclude that we can **STILL** not exclude...’.*

We confirm that we have read this submission and believe that we have an appropriate level of expertise to confirm that it is of an acceptable scientific standard.

# Peer review: Reviewer response for version 1

**DOI:** 10.5256/f1000research.19637.r44908

**Published:** 2019-03-19

**Authors:** Basil Donovan, Hamish McManus

**Affiliations:** 1 The Kirby Institute, The University of New South Wales (UNSW Sydney), Sydney, Australia; 2 The Kirby Institute, The University of New South Wales (UNSW Sydney), Sydney, Australia

This is a well written study aimed to measure correlation between country-level sexually transmissible infection (STI) screening intensity in MSM, and country-level incidence of *Neisseria gonorrhoeae* (NG) and *Chlamydia trachomatis* (CT) in MSM in European countries. The study sought to address a hypothesised association between risk of reinfection by unscreened cases and the intensity of screening in those populations. This hypothesis is consistent with no prior studies having detected negative correlation between screening and prevalence of STIs. While the study is ultimately unable to draw conclusions on the association between screening intensity and prevalence, its importance lies in potentially motivating discussion and further examination of this topic. One of the most important points it illustrates is that findings based on cross-sectional data such as these are open to a number of interpretations, and hence motivate more thorough analyses using longitudinal data.

 In part the reason for the fairly limited conclusions and presentation of a range of interpretations of the findings lies with limitations of the ecological source data. For example, the source data used for incidence estimation should be interpreted cautiously. The ECDC estimates cited are, precisely, national-level notification rates for the general male population rather than incidence rates for the respective MSM population. This is problematic for several reason. Firstly, the association between increased screening intensity and notification rates has been established by previous studies and estimates of incidence based directly on notification rates need to be adjusted accordingly to reflect this. ^1^^,^^2^ And, secondly, it is not made clear what effect the use of general male population ECDC estimates rather than MSM specific estimates has on results. Although authors suggest that the ECDC estimates are strongly weighted by MSM, this statement is not supported. While MSM are disproportionately represented in NG/CT notifications, it is still probable that the strength of correlation between screening in this population and general incidence rates could be reduced substantially given that MSM comprise a very low (<7%) proportion of the male population.

 In part to address these limitations (“To deal with this bias and the fact that the ECDC NG/CT incidence estimates do not provide incidence estimates for MSM” [P5]), the correlation between screening and prevalence estimates in MSM from STI clinics was also evaluated. However, this analysis is relatively limited, and we are not sure that it addresses these concerns successfully. Specifically, the prevalence results do not improve the interpretability of the incidence results. Also, they are based on complete results from only 4 countries (2 of which are not included in the incidence comparison) which is likely to limit the levels of correlation capable of being determined.

 To overcome the limitations of this ecological study, the author concludes that randomised controlled trials are urgently required. However, as this would require abandoning STI screening for some of the participants, such a trial would be ethically dubious and contrary to current clinical guidelines. Alternatively, longitudinal administrative data can be subject to retrospective cohort analysis. Using this technique, we were able to determine a true national increase in the incidence of NG in MSM but, after controlling for test frequency, this could be explained by increasing partner numbers and condomless anal sex. ^3^ 

 However, the statistical analyses in Kenyon’s study are appropriate and robust. More complex methods may have been inappropriate for the broad ecological source data used. Appropriately, the conclusions drawn are careful and, in that regard, supported by the results. This study should proceed to being indexed for the reason that it motivates discussion and should motivate more rigorous research into these growing epidemics.

We confirm that we have read this submission and believe that we have an appropriate level of expertise to confirm that it is of an acceptable scientific standard.

## References

[1] : Sexually transmitted infections in Europe 1990–2010. *ECDC*.2012; 10.2900/59547 10.2900/59547 Reference source

[2] : Nowhere near the point of diminishing returns: correlations between chlamydia testing and notification rates in New South Wales. *Aust N Z J Public Health*.2005;29(3) :249-53 1599177310.1111/j.1467-842x.2005.tb00763.x

[3] : Gonorrhoea gone wild: rising incidence of gonorrhoea and associated risk factors among gay and bisexual men attending Australian sexual health clinics. *Sex Health*.2018; 10.1071/SH18097 10.1071/SH18097 30409244
